# Gene expression in the context of malignant hyperthermia status and ageing

**DOI:** 10.1186/1471-2253-14-S1-A20

**Published:** 2014-08-18

**Authors:** Kathie Nicoll Baines, Dorota Fiszer, Philip Hopkins, Marie-Anne Shaw

**Affiliations:** 1 Malignant Hyperthermia Investigation Unit, St James’s University Hospital, LS9 7TF Leeds, UK

## Background

This study aims to investigate gene expression in skeletal muscle in relation to age and MH status, focussing on particular genes of interest. Expression patterns have been analysed to address the question of whether major causative malignant hyperthermia (MH) mutations influence skeletal muscle ageing. In this context we have examined gene expression in MH susceptible, both with (MHS+) and without mutation (MHS-), and MH normal (MHN) material from in vitro contracture test (IVCT) muscle biopsies.

## Material and methods and results

Affymetrix data, derived from Affymetrix HG_U133Plus2.0 arrays on a cohort of 59 patient muscle cDNA samples, were analysed for potential genes of interest. These 59 samples comprised an age range of 10-71 years and included 27 MHS+, 13 MHS- and 19 MHN. Linear models (LM) were applied using R studio 3.0.1©. LM1 incorporated three independent variables: age, sex and malignant hyperthermia (MH) status and one dependent variable, expression. LM2 included just the sex and MH status variables, LM3 included only the age and MH status and LM 4 included all 3 variables but left out the age-MH status interaction term. Model comparison was completed using Akaike Information Criterion to establish the model that best fitted the observed data. The results were interpreted to ascertain those genes that may be of interest in the context of age, MH status and interaction between these two variables.

The genes selected through this process, along with additional genes of interest based on criteria, such as involvement in store-operated calcium entry (SOCE) in skeletal muscle, were then subjected to TaqMan Gene Expression Analysis on a cohort of 108 cDNA samples (independent of the Affymetrix cohort) derived from patient muscle biopsies. This group comprised individuals with ages ranging from 10-87 years and represented individuals susceptible to MH, including those with a known mutation in *RYR1* (42) and those with no mutation in *RYR1* (22), and those with a normal phenotype (44). The results were analysed in a similar manner to the Affymetrix data, establishing any differences in gene expression according to the three dependent variables; age, sex and MH status.

## Conclusions

These results indicate a number of genes that may be of interest in relation to skeletal muscle ageing and MH status.

